# The Utility of Independent Component Analysis and Machine Learning in the Identification of the Amyotrophic Lateral Sclerosis Diseased Brain

**DOI:** 10.3389/fnhum.2013.00251

**Published:** 2013-06-10

**Authors:** Robert C. Welsh, Laura M. Jelsone-Swain, Bradley R. Foerster

**Affiliations:** ^1^Department of Radiology, University of Michigan, Ann Arbor, MI, USA; ^2^Department of Psychiatry, University of Michigan, Ann Arbor, MI, USA; ^3^Ann Arbor VA Healthcare System, Ann Arbor, MI, USA

**Keywords:** independent component analysis, support vector machine, resting-state functional connectivity, amyotrophic lateral sclerosis, machine learning, disease-state classification

## Abstract

Amyotrophic lateral sclerosis (ALS) is a devastating disease with a lifetime risk of ∼1 in 2000. Presently, diagnosis of ALS relies on clinical assessments for upper motor neuron and lower motor neuron deficits in multiple body segments together with a history of progression of symptoms. In addition, it is common to evaluate lower motor neuron pathology in ALS by electromyography. However, upper motor neuron pathology is solely assessed on clinical grounds, thus hindering diagnosis. In the past decade magnetic resonance methods have been shown to be sensitive to the ALS disease process, namely: resting-state connectivity measured with functional MRI, cortical thickness measured by high-resolution imaging, diffusion tensor imaging (DTI) metrics such as fractional anisotropy and radial diffusivity, and more recently magnetic resonance spectroscopy (MRS) measures of gamma-aminobutyric acid concentration. In this present work we utilize independent component analysis to derive brain networks based on resting-state functional magnetic resonance imaging and use those derived networks to build a disease state classifier using machine learning (support-vector machine). We show that it is possible to achieve over 71% accuracy for disease state classification. These results are promising for the development of a clinically relevant disease state classifier. Future inclusion of other MR modalities such as high-resolution structural imaging, DTI and MRS should improve this overall accuracy.

## Introduction

Amyotrophic lateral sclerosis (ALS) is a progressive neurodegenerative disease involving the motor cortex, corpus callosum, cortical spinal tract, and spinal anterior horn neurons, and presents with upper motor neuron and lower motor neuron signs (Ghadge et al., 2003; Turner et al., 2009). The disease can have a highly variable presentation and can be challenging to diagnose, which can have significant implications for the patients as the median survival time is between 2 and 4 years (Beghi et al., 2006). There is no definitive diagnostic test for ALS. The diagnosis relies on the clinical examination to detect upper and lower motor neuron signs in multiple body segments (Brooks et al., 2000) along with symptom progression. Unfortunately, there is on average a 1-year delay between onset of symptoms and diagnosis for this rapidly progressive disease (Zoccolella et al., 2006), which precludes timely intervention with emerging disease-modifying treatments. The development of reliable diagnostic and prognostic biomarkers would represent a significant advance in the clinical work-up of ALS (Karitzky and Ludolph, 2001; Cudkowicz et al., 2004; Turner et al., 2009).

Conventional magnetic resonance imaging provides limited and potentially inconsistent information describing ALS patients (Cheung et al., 1995; Hofmann et al., 1998; Comi et al., 1999; Chan et al., 2003). Therefore, there has been great interest in using advanced neuroimaging modalities to establish markers of ALS. Although techniques such as voxel-based morphometry (Roccatagliata et al., 2009), resting-state functional connectivity (Mohammadi et al., 2009; Jelsone-Swain et al., 2010; Verstraete et al., 2011; Agosta et al., 2013), magnetic resonance spectroscopy (Foerster et al., 2012a), and diffusion tensor imaging (Filippini et al., 2010) have demonstrated differences between groups of ALS patients and healthy controls (HC), few studies have investigated diagnostic test accuracy measures (Turner and Modo, 2010; Foerster et al., 2012b).

Functional connectivity is a relatively new and powerful advanced neuroimaging method to evaluate regional brain interactions (establishing neural networks) that occur when a subject is not performing an explicit task (Biswal et al., 1997; Lowe et al., 1998; Jelsone-Swain et al., 2010). Alterations of brain networks have been seen in diseases such as Alzheimer’s disease (Greicius et al., 2004), schizophrenia (Welsh et al., 2010), depression (Zeng et al., 2012), obsessive compulsive disorder (Stern et al., 2011), as well as ALS (Mohammadi et al., 2009; Jelsone-Swain et al., 2010; Verstraete et al., 2010, 2011; Douaud et al., 2011; Agosta et al., 2013). In particular, there is evidence of extensive brain network alterations due to the ALS disease process, such as those affecting the default-mode network (Mohammadi et al., 2009), motor networks (Douaud et al., 2011), and fronto-parietal networks (Agosta et al., 2013).

Statistical image analysis that can incorporate the entirety of a brain image can have an advantage over massively parallel univariate techniques (Wang and Summers, 2012). Machine-learning methods integrate a potentially large number of observables (that is, variables or features, and in the example of functional connectivity the feature space spans the number of connection strengths/edges derived from each resting-state time-series) into a coherent analysis that leverages the combined space of the features into an increase of detection power (Chen et al., 2008). Machine-learning methods using functional connectivity data have been applied to classify disease state such as in Alzheimer’s disease (Magnin et al., 2008; Orrù et al., 2012), depression (Craddock et al., 2009), and other psychiatric diseases (Orrù et al., 2012), and therefore could also be applied to ALS. To meet this important unmet need, we have explored the utility of machine-learning methodology to analyze resting-state functional magnetic resonance imaging (fMRI) data for ALS disease classification.

## Materials and Methods

### Participants

We recruited 32 patients diagnosed with ALS and 31 age and gender matched healthy controls (HCs). The ALS patients were recruited through the University of Michigan Motor Neuron Disease Clinic in the Department of Neurology at the University of Michigan. HC participants were recruited through local advertising and web portals. This study was approved by the University of Michigan Institutional Review Board. The participants gave informed consent prior to the MRI examination. All participants in this cohort underwent MRI examination, which included resting-state fMRI. All ALS participants had date of symptom onset recorded as well as disease severity at time of scan assessed by the ALS Functional Rating Scale, revised version (ALSFRS-R) (Cedarbaum et al., 1999). The maximum score of the ALSFRS-R is 48, with lower scores indicating increased physical disability.

### Magnetic resonance acquisition

#### Image acquisition

All scanning took place on a GE 3T Excite 2 magnet (General Electric, Milwaukee, WI, USA). All participants had high-resolution anatomic T_1_-weighted imaging (spoiled-gradient-recall, SPGR). High-resolution images were collected with a 256^2^ matrix, 220 mm FOV, and 1.0 mm slice thickness) and resting-state fMRI. T_2_^∗^-time-series data were acquired parallel to the AC-PC axis using a reverse-spiral *k*-space readout. A total of 240 T_2_^∗^-weighted volumes were collected during each scanning session (repetition time, TR = 2 s; 40-slice volumes; 3 mm slice thickness, no skip; echo time, TE = 30 ms; 64 × 64 matrix; field-of-view FOV = 220 mm).

#### Functional connectivity

Resting-state time-series data were pre-processed similarly to Welsh et al. (2010). We used an in-house pre-preprocessing method which uses both FSL 4.1.9 (Jenkinson et al., 2012) and SPM8 (release 4667). Time-series data were preprocessed in the following steps: slice-time corrected (FSL), motion corrected (FSL), and normalized to MNI space (SPM8/VBM8). Time-series data were resampled to 3 mm voxel resolution and isotropically smoothed with a 5-mm Gaussian kernel. A mask of white-matter was derived from the SPGR during the spatial normalization step using VBM8. To minimize partial volume effects, the resulting mask was eroded three times over with FSL. A similarly derived cerebral spinal fluid (CSF) mask was also created, however, due to variance in ventricular size across subjects the CSF mask was only eroded once. Prior to independent components analysis (ICA) data were further filtered: (1) global signal normalization was performed (Chang and Glover, 2009; Fox et al., 2009); (2) motion parameters (translation and rotation) were regressed from the time-series data; (3) voxel time-courses were then extracted from white-matter and CSF masks and analyzed with principle components analysis (PCA), following Behzadi et al. (2007) the top five PCA components were then used to regress out systematic variance due to physiological noise; (4) data were then band-pass filtered (fast-Fourier transform) in the 0.01–0.10-Hz range (Cordes et al., 2001).

The resulting time-series data for each subject was then independently analyzed with ICA using FSL/Melodic (Beckmann and Smith, 2004). The number of components was not specified as the number was best determined by Melodic (Beckmann and Smith, 2004) using the Minimum Description Length algorithm (Rissanen, 1978). The ICA analysis produced between 15 and 40 ICA spatial components and corresponding temporal modes^1^.

Next, we used the spatial templates from the networks defined in Smith et al. (2009). We took the top 10 templates defined from their BrainMap analysis^2^ in the 20-component ICA scheme, thresholding each map at (component magnitude)>3.0. These template network maps were then used to identify the corresponding resting-state network (RSN) in our analysis. Assignment of a particular network to a component was done by maximizing the overall match for all 10 RSNs following the procedure of Greicius et al. (2004). A score was calculated for each network for the best matching ICA spatial component by taking the average of the in-map spatial component weight minus the average out-of-map spatial component weight. We required that a component could only be used once and if there was one component best matched to two or more RSNs, then all possible combinations were searched to get an overall best RSNs match for that subject.

In order to provide properly scaled data to the support vector machine, a correlation map for any particular RSN was created by calculating the correlation coefficient for each voxel in the RSN with the associate ICA time-course, after all other ICA time-courses had been regressed from the voxel time-series.

**Figure 1:**
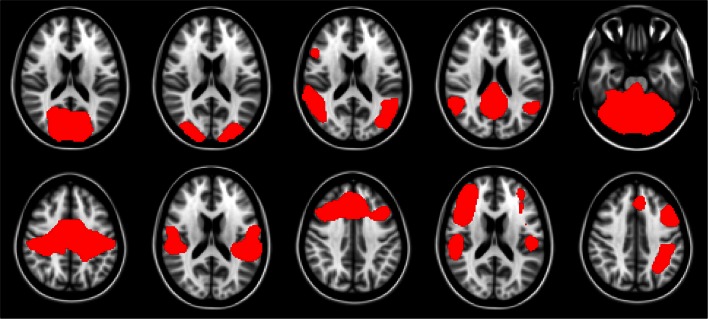
**Resting-state network templates corresponding to networks 1 through 10 from Smith et al. (2009)**. RSNs are numbered 1 through 10, with upper left being 1, and lower right be number 10. As in Smith et al. (2009) networks are as follows: 1–3: visual, 4: default mode, 5: cerebellum, 6: sensorimotor, 7: auditory, 8: executive, 9–10: fronto-parietal.

For this work we explored the utility of disease state classification based upon the networks that have been shown to be altered in ALS: DMN, Motor, and Fronto-Parietal. Additionally, given the observed ∼35% cognitive impairment in ALS (Jelsone-Swain et al., 2012) we also included the frontal executive network. In the Smith et al. (2009) nomenclature these are RSNs: RSN04, RSN06, RSN07^3^, RSN08, RSN09, RSN10. The selected networks are shown in Figure 1.

### Support vector machine

Current implementations of support vector machines were first formulated by Cortes and Vapnik (1995). Briefly, a support vector machine is a supervised learning formalism that allows for complex solutions of discrimination classification. Typically an array of measures is carried out for each instance of a class. In our study measures of connectivity (the array of measures) are determined for each participant (each participant being an instance/observation) in the study. Unlike a massively parallel univariate analysis carried out between two groups, support vector machine formalism examines all measures simultaneously to determine a hyperplane in the space defined by the array of measures that may separate the two groups. Using the notation of Guyon and Elisseeff (2003):

D(x)=wx+b

using a training data set, the decision boundary is optimized to give the weights ***w***. At the testing phase the decision solution for a given observation **x** of the array of measures can be calculated with the weight vector ***w*** determined by the SVM, and *b* is a bias value also determined by the SVM. When *D*(**x**) < 0, then **x** belongs to the first class, while *D*(**x**) > 0 indicates membership in the second class. *Inherently, the decision is a binary one*. Further formalism of the SVM methodology can be found in Cortes and Vapnik (1995).

**Figure 2:**
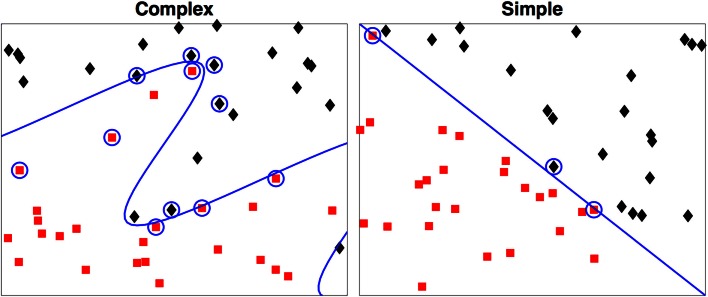
**Two-dimensional examples of a simple and a complex support vector machine solution**. The two-class membership is indicated by color. The support vectors are indicated by the circles with the boundary defined by *D*(**x**) = 0. During testing only the support vectors are used in determination of the class for the test case.

As a simple example we illustrate this concept with Figure 2. In both examples each observation is characterized by two metrics. The boundary of the first is easily derived, but the boundary of the second that maximally discriminates between the two groups can take a highly complex form, even in this two-dimensional example.

We utilized the support vector machine (*libsvm version 3.17*) implementation of Chang and Lin (2011) and we opted to use the linear kernel. Given the observations in the literature for compromises related to ALS in a variety of resting-state networks, we built feature-vectors of correlation coefficients from the following RSNs: default mode (Mohammadi et al., 2009), motor areas (Verstraete et al., 2010, 2011; Douaud et al., 2011), and left and right frontal-parietal regions (Agosta et al., 2013).

**Figure 3:**
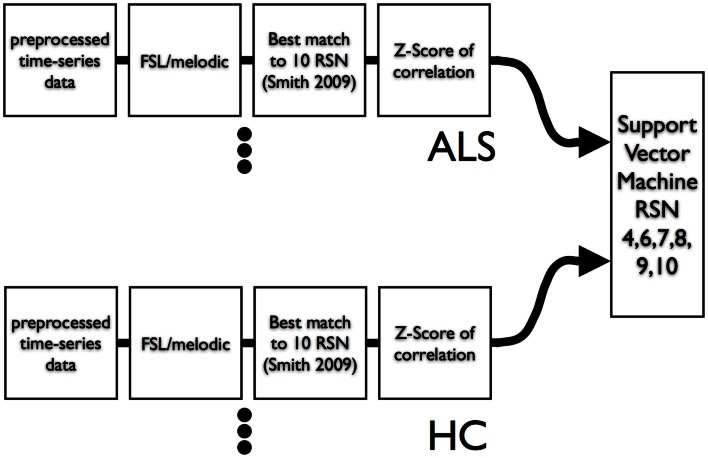
**Data flow of resting-state times series into ICA and then into SVM**. A leave-one-out-cross-validation was utilized to assess SVM classification accuracy.

We used leave-one-out-cross-validation (LOOCV) (Burges, 1998) for calculation of SVM accuracy. The overall scheme is shown in Figure 3. We also examined the efficacy of simple feature filtering by including those features from a network that passed a two-tailed liberal statistically significant group difference of *p* ≤ 0.05 (uncorrected) (Craddock et al., 2009). We performed a bootstrap (Jiang and Simon, 2007) on the LOOCV (100 bootstraps) and calculated the class prediction for each test case in the LOOCV as a continuous variable by averaging the 100 binary decision results. This average class prediction then allowed for the calculation of a receiver-operator curve (ROC). To estimate the variance on the area-under-the-curve (AUC) of the ROC we performed a bootstrap ROC.

Final classification accuracy was defined as the:

$$\frac{N_{\text{Correct}}^{\text{ALS}}+N_{\text{Correct}}^{\text{HC}}}{N_{\text{Total}}^{\text{ALS}}+N_{\text{Total}}^{\text{HC}}}$$

with $N_{\text{Total}}^{\text{ALS}}=32$ and $N_{\text{Total}}^{\text{HC}}=31$. $N_{\text{Correct}}^{\text{ALS}}$ and $N_{\text{Correct}}^{\text{ALS}}$ being the number of correctly SVM classified ALS and HC participants. To assess performance of the SVM against a typical univariate method, we followed methods by Fair et al. (2007) and calculated the number of nodes (Sporns, 2009) (voxels) present for a subject in the given network surpassing a *Z*-score of 0.10, 0.15, 0.20, 0.25, and 0.30. The number of nodes by subject was then used to calculate a univariate ROC.

## Results

### Demographics

**Figure 4:**
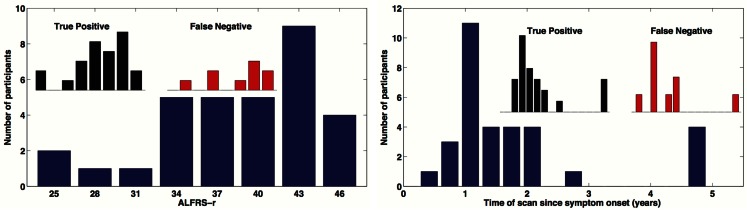
**Distribution of time of scan since symptom onset and observed distribution of ALSFRS-r in our ALS participant cohort**. Inserted distributions are for the true-positive and false-negative classified groups.

A total of 32 individuals with ALS were enrolled in our study. Our main objective for HCs was to match for age. Mean ALS age was 58.4 ± 6.6 years and our HCs were aged 56.9 ± 5.0 and there was no significant difference in age (two-sample *t*-test *p* = 0.319). We did have a slight imbalance in gender matching, with ALS male/female = 21/11, and HC male/female = 16/15. However there was no age by gender bias, *p* > 0.05. Mean time since onset of symptoms for ALS was 1.8 ± 1.4 years with a range of 0.4–6.0 years. ALSFRS-R average score was 38.2 ± 5.7 with a range of 25–46. The ALSFRS-R score and time of scan since symptom onset distributions are show in Figure 4.

### ICA group validation

**Figure 5:**
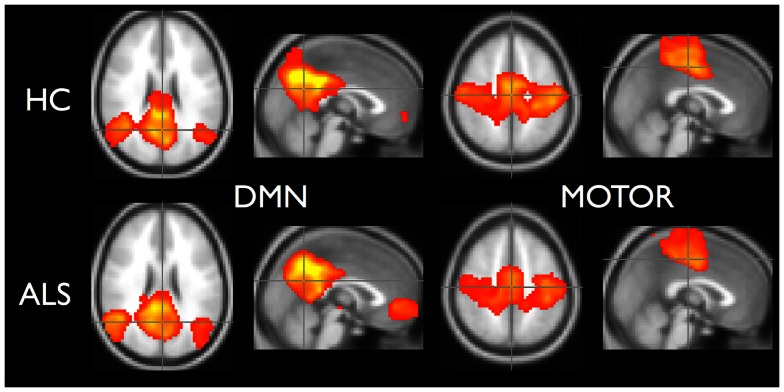
**Random effects analysis for default-mode network and motor network**. Healthy controls are in top row and ALS are in bottom row. Statistical maps thresholded for *t* ≥ 3.5 for illustrative purposes.

To demonstrate that the template matching succeeded, we calculated typical resting-state group analyses: subject correlation maps for each RSN were converted to *Z*-scores and entered into random effects analyses by group. Statistical images for the default mode (RSN04) and the primary motor network (RSN06) are shown in Figure 5.

### Support vector machine results

**Figure 6:**
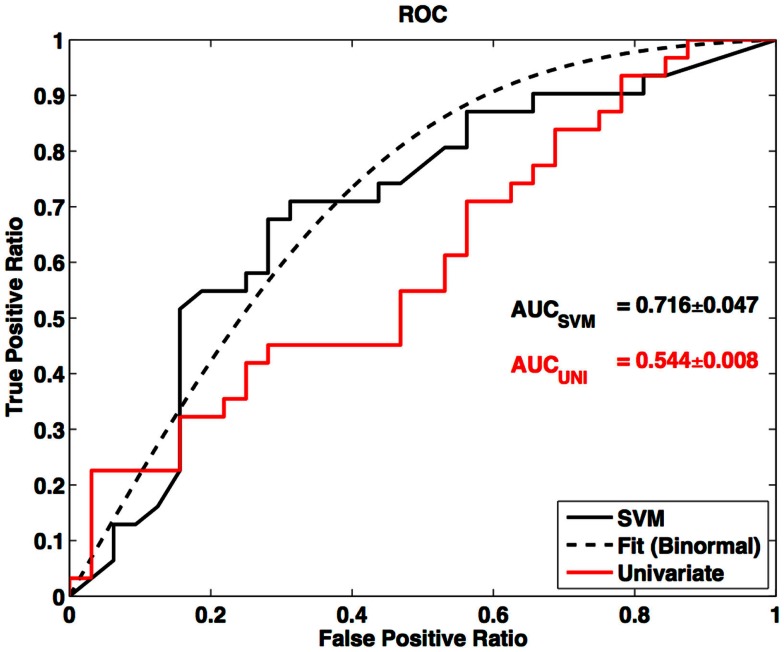
**Receiver-operator curve for LOOCV SVM and ROC for univariate node counting**. Smooth curve is a binormal (Cai and Moskowitz, 2004) fit to the LOOCV SVM ROC. AUC is calculated from binormal fit.

In this survey of classification performance the SVM achieved 71.5% accuracy for determination of disease state as either ALS or healthy. This maximal classification accuracy came from a combined use of the default-mode network (RSN04) and the primary motor network (RSN06). For this combination the fraction of correctly classified ALS and HC was $N_{\text{Correct}}^{\text{ALS}}=23$ and $N_{\text{Correct}}^{\text{HC}}=22$. The SVM classification and univariate classification ROCs are shown in Figure 6. The bootstrap calculated AUC and variance was AUC = 0.716 ± 0.047. The univariate AUC was AUC = 0.544 ± 0.008. To test for a classification bias due to ALSFRS-R or time-of-scan-since-symptom-onset (ONSET) we did a *post hoc* examination of the ALSFRS-R score and ONSET for those ALS participants that were accurately classified as ALS and those incorrectly classified as healthy. We also tested disease progression rate [defined as (48-ALSFRS-R)/ONSET] between these groups. We performed a non-parametric Kolmogorov–Smirnov (Chakravarti et al., 1967) test to assess if these values were drawn from the same or different parent distribution. Comparison of ALSFRS-R, ONSET, and progression rate between true-positive and false-negative groups revealed no significant differences (*p* = 0.306, *p* = 0.744, and *p* = 0.372 respectively). The ALSFRS-R and ONSET distributions for true positives and false negatives are shown in Figure 4.

## Discussion

Our work combined resting-state connectivity [derived from ICA (Beckmann and Smith, 2004)] and machine learning (Chang and Lin, 2011) to explore their utility for ALS disease-state prediction. ICA reliably identified well established (Damoiseaux et al., 2006; Smith et al., 2009) RSNs in our ALS and HC cohorts. By using a subset of these networks that have been shown to be altered by the ALS disease process (Mohammadi et al., 2009; Jelsone-Swain et al., 2010; Verstraete et al., 2010; Douaud et al., 2011; Agosta et al., 2013), in conjunction with machine learning (as implemented with a support vector machine), we have shown that machine learning has modest disease classification accuracy using resting-state fMRI data. The AUC of the SVM indicates better performance than the univariate classifier. In schizophrenia SVM derived classifications have been found to be between ∼62 and ∼85% (Tang et al., 2012; Yu et al., 2013) using whole brain connectivity. Using a specific and more finite number of network nodes Craddock et al. (2009) achieved 62% classification accuracy in depression with comparable *t*-test filtering.

Although extra motor regions have been implicated in ALS using other advanced neuroimaging methods such as diffusion tensor imaging (DTI), it is important to note that the motor networks had significant contribution to the SVM state classification. Though not presently recognized as a resting-state network showing alteration, the executive control network also contributes to the disease state classification. The classification sensitivity to the executive network could be due to the ∼35% observed cognitive impairment seen in ALS (Rippon et al., 2006; Jelsone-Swain et al., 2012). Our findings demonstrate the power of multivariate techniques such as machine learning. We have shown there can exist significant systematic differences when the network is considered as a whole even though there can be a lack of statistically significant differences in specific node or edge-wise comparisons, such as in counting of significant nodes in a network (Sporns, 2009).

Seeley et al. (2009) suggested that disease state can be classified by functional network metrics derived from resting-state measurements using fMRI. Until recently the vast majority of the resting-state fMRI literature tested for statistically significant group differences with a voxel-wise approach (Smith, 2012). Much of that work was done with either seed based analysis or ICA (Fox et al., 2005; Damoiseaux et al., 2006) to determine these brain networks. The voxel-wise approach requires a spatial coherence in change due to the disease process, but also requires an observable change that meets statistical significance after correction for multiple comparisons (Marchini and Presanis, 2004). Although in our previous approach of investigating group differences, we relaxed this condition of spatially coherent change in the network by examining distribution differences of network metrics (correlation coefficient) (Jelsone-Swain et al., 2010). More recent group comparison approaches have taken on graph theoretical methodology (Cabral et al., 2011) to address questions of group differences.

In a multivariate approach, the data are used coherently to assess significance between groups. This approach is readily extended to build a decision algorithm to determine group membership based on the full suite of variables under consideration. The decision algorithm can be trained with an independent dataset and then assessed for accuracy through the use of an independent testing dataset. Indeed this is the operational approach of machine learning (Vatolkin et al., 2012). Essentially a mathematical decision boundary can be derived in the space of the suite of variables. This boundary is derived to maximize the separation of the groups to be classified [though due to noise in real systems one would not expect 100% separation (Wang and Summers, 2012)].

Discovering differences in brain metrics between two cohorts leads to a better understanding of the effect that a disease process has on a brain (Bandettini, 2012), such as an aberration in the motor network in ALS (Jelsone-Swain et al., 2010; Verstraete et al., 2010, 2011; Douaud et al., 2011). However, with increased understanding of observed changes, patterns can be revealed. These group differences can manifest patterns that can then be identified through machine-learning algorithms, more specifically in regard to identifying group membership. Eventually this can lead to the use of brain metrics to classify between two brain activity states (Laconte et al., 2005; De Martino et al., 2008; LaConte, 2011), or between states of diseased and healthy (Orrù et al., 2012). Thus, invoking multivariate techniques can lead to better state differentiation than differentiation based on voxel-wise or edge-wise univariate comparisons.

Advanced neuroimaging techniques, specifically resting-state fMRI, DTI and voxel-based morphometry, generate a large number of potentially useful data points. It is becoming increasingly clear that more conventional univariate brain analysis techniques used in concert with single modality imaging techniques do not provide sufficient disease discrimination in ALS. For example, a meta-analysis of DTI data results indicates only modest diagnostic test accuracy in ALS (Foerster et al., 2012b). As a result there is increased interest in applying more advanced brain mapping statistical techniques in ALS, including the implementation of machine-learning methods to analyze advanced MRI data in an effort to develop an imaging “fingerprint” of disease. The results presented here point to the potential utility of machine-learning methods to classify disease status in ALS using imaging data sets with a large number of variables. Additional research efforts are required to further explore this approach including combining different advanced neuroimaging approaches using machine-learning methods. In addition to resting-state fMRI the modalities to be included should be: DTI (Chapman et al., 2013), high-resolution structural imaging (Grosskreutz et al., 2006), and magnetic resonance spectroscopy (Foerster et al., 2012a) as put forth by Turner et al. (2009). Furthermore, given the heterogenous presentation of ALS, which can lead to clinical diagnostic uncertainly, it would be warranted to apply SVM classification methods and advanced neuroimaging techniques to a cohort of individuals at first presentation with neurological symptoms. The imaging should occur prior to a definitive diagnosis of ALS and follow the individuals longitudinally. Given the differential nature of disease diagnosis, future studies should also include ALS mimics, that is, other neurodegenerative diseases with overlapping symptom presentation, to fully explore the power of classification schemes (Turner and Modo, 2010). The true diagnostic utility of such a classifier would be in providing input into the clinical process with the resulting goal of shortening the duration between symptom presentation and final diagnosis of ALS.

### Limitations

There are of course limitations to our study. First, ALS is a highly divergent disease process with highly varying progression paths. Certainly, utilizing a larger cohort of individuals with ALS and a larger cohort of HCs would lead to a better definition of classifiers. Though the ALS disease process has a quite divergent nature we have built our classifier decision from two classes. Another approach would be to build a single state (one-class) classifier (Manevitz and Yousef, 2002). Under those conditions, the question would be, “*Does the test case belong to the one-class classifier?*” Our approach has also only included a single category of metrics, namely resting-state derived brain networks. Other MRI modalities have also been shown to be sensitive to the ALS disease process, such as cortex thinning (Roccatagliata et al., 2009; Turner et al., 2009), increased radial diffusivity (RD) and decreased fractional anisotropy (FA) (Wang and Melhem, 2005; Schimrigk et al., 2007; Filippini et al., 2010; Foerster et al., 2012b), and more recently decreased gamma-aminobutyric acid (GABA) concentration in the motor cortex (Foerster et al., 2012a). We do note that this is the first application of machine learning for the classification of disease status in ALS using MRI data^4^. Construction of a complex differential diagnosis classification scheme first has to demonstrate distinction between well-defined classes such as definite ALS and HCs. As such, this current work is exploratory in nature but demonstrates the clear promise of such techniques to continue in the near future.

## Conclusion

Resting-state functional connectivity reveals intrinsic networks in the human brain. These networks can be viewed as patterns that are a manifestation of the state of the brain including altered network patterns present in disease (Seeley et al., 2009). By applying multivariate pattern classification methodology we have demonstrated that machine-learning methodology (support vector machine) in conjunction with brain networks derived from resting-state fMRI can be used to classify a diseased brain (ALS) from a healthy brain. Additional research efforts are required to validate our findings as well as to investigate the added diagnostic utility of including other MR modalities in the setting of ALS using machine-learning methods.

## Conflict of Interest Statement

The authors declare that the research was conducted in the absence of any commercial or financial relationships that could be construed as a potential conflict of interest.

## Footnotes

^1^Mean number of components for ALS was 27 +/−7 and the mean number of components for HC was 24 +/- 4.

^2^http://www.fmrib.ox.ac.uk/analysis/brainmap+rsns/

^3^We visually inspected each RSN template prior to selection. By using the “atlas” tool in FSL we determined that RSN07 also included portions of the motor system (pre-central gyrus), therefore we included it as a relevant network.

^4^A pubmed.org search of the title/abstract terms [(“ALS” or “amyotrophic lateral sclerosis”) and (“support vector machine” or “SVM”)] yields no imaging literature.
